# Transportation of Sick and Wounded

**Published:** 1918-09

**Authors:** F. S. Gallie


### TRANSPORTATION OF THE WOUNDED

**Transportation of Sick and Wounded.** Colonel F. S. Gallie, R. A. M. C.

The transportation of sick and wounded from the front area to the hospital bases in France and from those bases to the United Kingdom presents, the speaker said, very many difficulties. He said he would deal especially with the transportation of patients to the bases, and of some of them eventually to England.

Plying between the casualty clearing stations and the various bases are ambulance trains and ambulance canal barges. The vast majority of patients are, of course, conveyed by ambulance train, and for this purpose there are, at present, no fewer than 41 such trains in commission.

### *Ambulance Trains.*

The growth of the ambulance train services will be readily appreciated when it is noted that although there were only 4 trains in August, 1914, 9 in October, and 12 in December of that year (one of which was of British stock), by the end of 1915 there were 20 trains in circulation, by the end of 1916, 30 trains, and by the end or 1917, 38 trains; whilst at the present time there are, as already stated, 41; of these 4 are reserved for service with the British Forces in Italy.

The earlier ambulance trains were primitive when compared with the later trains which were built in England, and which are so furnished and equipped as to constitute mobile hospitals.

In the earlier trains, which were of necessity composed of French railway (non-communicating) rolling stock, patients were largely carried on field stretchers suspended on Brechot frames. On these improvized trains the culinary facilities were not great, the sanitary arrangements were improvized, and it was only by the exercise of marked initiative on the part of the train commanders that the success which crowned the work of these earlier units was won.

In March, 1915, the 11 French trains then in our service were re-constituted, and their carrying capacity greatly increased; internal alterations of the coaches added to the comfort of the patients carried, and many other facilities were provided, which increased the efficiency of the train service generally.

Each train added to the service since December, 1914, is composed of British rolling stock, and the present day ambulance train leaves little to be desired as regards facilities for the treatment of patients *en route* from the forward areas to the bases, or during the long journeys from Italy and from Marseilles to the English Channel ports.

An ambulance train generally comprises: 9 coaches for wounded (each coach containing from 36 to 42 bed cots); 1 coach fitted as dispensary and operation room; 1 as isolation ward; 2 as kitchen coaches; 1 as stores coach; with 1 coach for the medical officers and nursing sisters and 1 for the personnel: that is, each train has 16 coaches, all intercommunicating, and is of a total length of about 320 yards. Each train accommodates some 500 patients, dependent on the numbers "lying" and "sitting", but in periods of marked activity up to 600 patients are frequently carried.

The United States military authorities have had a large number of ambulance trains constructed in England, and these are now in commission in this country. The model of the British train was accepted as suitable, and the U. S. trains as they run, are practically identical with the British trains. The speaker thought it very advantageous that this should be the case, as it makes the respective service of ambulance trains homogeneous. The trains can be interchanged, and already the respective medical services have helped one another by such interchange during periods of activity in particular areas.

The importance of making trains as comfortable as possible for the carriage of the grievously wounded cannot be exaggerated. The ambulance train is the first medical unit in which the wounded patients feel that they are really resting. They have left the noise of the guns, the turmoil and tumult of the battle area, and have started on the road to recovery, and it is of the utmost importance that every effort be strained to provide all the comforts, and even luxuries, possible, for the benefit of those who have fought heroically, and to whom fate has allotted the pain and discomfort of battle wounds.

In periods of expected activity in any area, empty ambulance trains are held in reserve in near garage stations. When the numbers of patients in the casualty clearing stations demand it, the Director of Medical Services of the army concerned asks that the necessary trains should be moved forward to the " loading point " and this is arranged for by the French railway authorities through the liaison of the British Railway Transport Department.

As the loaded train leaves the railhead for the hospital base, the detail of the convoy and the hour of its departure is notified by telegram to the Administrative Medical Officer of the Base to which it is consigned, and to the Director of Medical Services, Lines of Communication.

Whilst en route to the base, such wounds as require dressing are attended to, splints are re-adjusted, medicines are given as required, and all patients are liberally fed and given comforts; in short, the patients whilst on the trains are treated, as far as facilities permit, exactly as in a base hospital.

On arrival of the trains at the base, stretcher-bearers are found waiting on the platform to detrain the patients, and motor ambulance cars in attendance for the conveyance of the patients to the various institutions at the hospital center.

The distribution of patients from the front areas is a matter of the greatest importance. The Headquarters office of the D. M. S., L. of C., decides the destination of each train in accordance with the numbers of vacant beds existing at the various hospital centers, always keeping in view the desirability of shortest possible journeys for severe cases. These destinations are given by him to the Director of British Railway Transport who, in turn, arranges with the French authorities that each train proceeds to the base allotted.

The telegraphic notification of the departure of the train from the railhead station enables the Administrative Medical Officer at the base to which the train is consigned to make the necessary arrangements for the reception of the patients on arrival, and to arrange that the necessary bearers and ambulance vehicles are actually awaiting the train as it arrives at the station.

Picture then these 41 trains — each capable of accommodating 600 patients — forming in truth a huge mobile hospital of 25,000 beds. An Army engaged in battle activity demands a section of this mobile hospital to be sent at once to the forward area to provide urgently required hospital accommodation for casualties. In accordance with that demand two, three, or even ten trains (each train in itself a complete hospital section) steams off to the scene, and takes over the patients.

It may happen that there is activity in two armies, or in four armies, but the trains are ample, and off steam the mobile hospital sections — complete not alone in medical equiment, but in medical personnel, supplies, stores, etc., — to relieve the congestion of the forward area hospitals by transferring the suffering, and that with all possible practical comfort, to the commodious and thoroughly equipped hospitals at the various bases.

Such a picture will give an idea of the ambulance train organization, an organization which employs some 100 medical officers, 150 nursing sisters, and over 2,000 rank and file.

*Stores and Garages.*

At certain of our large bases we have garage stations for such ambulance trains as are being held in reserve and at these stations are established train supply stores at which establishments all ambulance trains are replenished with necessary food, comforts, linen, ordnance equipment, etc.

Small workshops are attached to the stores, and facilities are afforded for minor repairs, etc., as are required by the trains.

*Barge Transport.*

As many waterways exist, particularly in Northern France, the transport of wounded men from certain of the forward areas to the bases by ambulance barges is possible, and our service of ambulance barges has been in existence since 1915.

The barges travel in pairs towed by a steam tug. Each barge is fitted as a hospital ward of 30 beds, and is specially equipped for the carrying out of necessary treatment. Such transport is particularly suitable for G. S. wounds of chest, head and abdomen, and indeed for very severe cases generally, but it has the definite disadvantages of being slow, being very limited in its capacity, and in its possibilities of application.

Our barges are very well fitted, they are all lighted with electricity and are kept in commission throughout the year.

They work under practically the same routine as do the trains and are of course under the same direction.

If any officers of the United States Army are specially interested in the detail of any particular branch of ambulance transport, he will gladly be afforded opportunities of seeing the system in being.

The speaker wished strongly to impress upon his hearers the necessity for realizing that the patients in the front area medical units require to be transported to the base by Ambulance trains, that it is essential to be practical in preparations especially with splints; although extreme abduction of fractured limbs may be desirable, the patient will have to be transported. The speaker had seen patients sent for transport, who, owing to the nature of the splints applied, could not be got through the double doors of the railway carriages.

Patients suffering from Compound Fractures of the lower extremities, and those suffering from severe chest and abdominal injuries should not be moved from the stretchers on which they are placed in the front area hospital, and a distinctive instruction to this effect should be given when transport begins; the bed cots on the trains being so constructed that stretchers can be laid on them and patients left undisturbed.

Patients should be carefully classified, and patients who are "in extremis" should not be sent to the trains.

As a general rule severe cases should be those first sent for entraining; this permits the train commander to assign to them the best cots. See that helpless patients are catheterized before being entrained — the train is a busy unit during the earlier stages of the journey.

*Ambulance Ships.*

From certain of the seaports in Northern France, ambulance transport ships sail to England. Such patients as are not considered likely to be fit for duty with the army, or for discharge from hospitals to the convalescent depots in this country within four to six weeks are embarked on these ships and by them transported to England, where, on arrival, they are distributed to various military hospitals for further treatment.

Some of our hospital centers are, as you know, established in the immediate vicinity of the seaports, and the selected patients from such centers are transferred direct to the ambulance ships by motor ambulances.

From the earliest day of the war the service has been continuous, and though some of our ships have fallen victims to enemy action the service has never for a moment halted.

"Carry On" has been the motto — and our sick and wounded soldiers fully appreciate the splendid spirit which has prompted their sailor brothers to continue the service of Cross-Channel transport, despite the inhuman attempts of the enemy to destroy, if possible, the vessels conveying the invalids to their homeland.

